# Second-order QCD corrections to event shape distributions in deep inelastic scattering

**DOI:** 10.1140/epjc/s10052-019-7528-3

**Published:** 2019-12-19

**Authors:** T. Gehrmann, A. Huss, J. Mo, J. Niehues

**Affiliations:** 1 Physik-Institut, Universität Zürich, Winterthurerstrasse 190, 8057 Zürich, Switzerland; 2 Theoretical Physics Department, CERN, 1211, Geneva 23, Switzerland; 3 Institute for Particle Physics Phenomenology, Durham University, Durham, DH1 3LE UK

## Abstract

We compute the next-to-next-to-leading order (NNLO) QCD corrections to event shape distributions and their mean values in deep inelastic lepton–nucleon scattering. The magnitude and shape of the corrections varies considerably between different variables. The corrections reduce the renormalization and factorization scale uncertainty of the predictions. Using a dispersive model to describe non-perturbative power corrections, we compare the NNLO QCD predictions with data from the H1 and ZEUS experiments. The newly derived corrections improve the theory description of the distributions and of their mean values.

## Introduction

Event shape variables allow various kinematical properties of hadronic final states to be analysed. The resulting event shape distributions were measured extensively in $e^+e^-$ [1] and *ep* [2] collisions, enabling a variety of precision QCD studies, including measurements of the strong coupling constant, resummation and parton-shower effects, investigations of non-perturbative power corrections, and tuning of multi-purpose event simulation models.

Precision studies of event shapes distributions demand that their theoretical description is of comparable accuracy to the experimental measurements, requiring the calculation of higher order contributions in perturbative QCD. For $e^+e^-$ event shapes, an appropriate level of theory precision was achieved already some time ago with the calculation of the next-to-next-to-leading order (NNLO) QCD corrections [3–9] in the form of generic parton-level event generators that allow any infrared-safe event shape distribution to be computed. These fixed-order NNLO results can be combined with resummation of large logarithmic corrections to next-to-next-to-leading logarithmic level (NNLL) and beyond for specific event shape variables [10–15].

For event shapes in deeply inelastic *ep* scattering (DIS), the currently available level of theoretical accuracy is lower, with fixed-order results only known to next-to-leading order (NLO) [16–18] and resummation at next-to-leading logarithmic level (NLL) [19–22]. The theory uncertainty (as quantified through variation of the renormalization and factorization scales) on these predictions is often comparable to or larger than the experimental errors on the event shape measurements from H1 [23] and ZEUS [24], thereby limiting the extraction of fundamental QCD parameters from these data. To overcome this limitation requires an improvement of the fixed-order predictions to NNLO, which is presented in the following.

This paper is structured as follows. In Sect. 2, we summarise the definitions of the most common DIS event shape variables, and the kinematical ranges covered by the H1 [23] and ZEUS [24] measurements. The calculation of NNLO corrections to event shape distributions is performed in the $\hbox {NNLO}{{\textsc {jet}}}$framework [25] and follows closely the related NNLO calculations of jet production in DIS [26, 27] and is documented in Sect. 3. To compare the resulting parton-level NNLO predictions with experimental hadron-level data, we employ a dispersive model [28–30], described in Sect. 4, determining the non-perturbative power corrections to the event shape distributions. We perform detailed comparisons of the hadron-level predictions to event shape data from H1 and ZEUS in Sect. 5. Our findings are summarized in Sect. 6.

## Event shape variables

Event shapes in DIS are measured in the Breit frame, defined by the momentum directions of the virtual photon (current axis) and the proton (remnant axis), and boosted such that the energy component of the virtual photon momentum vanishes. The Breit frame provides a separation in pseudorapidity $\eta$ between the proton remnant (remnant hemisphere, $\eta >0$) and the hard scattering process (current hemisphere, $\eta <0$). The event shape variables are dimensionless quantities that are determined from the four-momenta $p_h= (E_h,\mathbf {p}_h)$ of all particles in the current hemisphere. The different variables [22], which are generically denoted as *F*, are defined as follows.

The thrust $\tau _\gamma$ measures the longitudinal momentum components projected onto the current axis:

1 $$\begin{aligned} \tau _\gamma = 1-T_\gamma , \quad \mathrm {with} \quad T_\gamma = \frac{\sum _h |{p}_{z,h}|}{\sum _h |\mathbf {p}_h|}\,. \end{aligned}$$

Thrust $\tau _T$ is the thrust with respect to the thrust axis in the direction $\mathbf {n}_T$ which maximizes the longitudinal momentum components projected onto this axis:

2 $$\begin{aligned} \tau _T = 1 - T_T, \quad \mathrm {with} \quad T_T = \max _{\mathbf {n}_T}\frac{\sum _h |\mathbf {p}_h \cdot \mathbf {n}_T|}{\sum _h |\mathbf {p}_h|}\,. \end{aligned}$$

This is analogous to the definition of thrust in $e^+ e^-$ collisions.

The jet mass parameter $\rho$ is the squared invariant mass in the current hemisphere, normalized to four times the total energy squared:

3 $$\begin{aligned} \rho = \frac{(\sum _h p_h)^2}{(2 \sum _h E_h)^2}\,. \end{aligned}$$

The jet broadening $B_\gamma$ measures the sum of the transverse momenta with respect to the current axis:

4 $$\begin{aligned} B_\gamma = \frac{\sum _h |\mathbf {p}_{t,h}|}{2 \sum _h |\mathbf {p}_h|}\,. \end{aligned}$$

As with thrust, the jet broadening can also be defined with respect to the thrust axis:

5 $$\begin{aligned} B_T = \frac{\sum _h |\mathbf {p}_h \times \mathbf {n}_T|}{2\sum _h |\mathbf {p}_h|}\,. \end{aligned}$$

Finally, the *C*-parameter is derived from the linear momentum tensor $\Theta ^{ij}$:

6 $$\begin{aligned} \Theta ^{ij} = \frac{1}{\sum _h |\mathbf {p}_h|} \sum _h\frac{ p_h^i p_h^j}{|\mathbf {p}_h|}\,. \end{aligned}$$

with the eigenvalues $\lambda _1, \lambda _2, \lambda _3$ of $\Theta ^{ij}$ yielding

7 $$\begin{aligned} C = 3(\lambda _1 \lambda _2 + \lambda _2 \lambda _3 + \lambda _3 \lambda _1)\,. \end{aligned}$$

Equivalently, it can be expressed as

8 $$\begin{aligned} C = \frac{3}{2} \frac{\sum _{h,h'} |\mathbf {p}_h| |\mathbf {p}_{h'}| \sin ^2 \theta _{hh'}}{(\sum _h |\mathbf {p}_h|)^2}\,, \end{aligned}$$

where $\theta _{hh'}$ is the angle between particles *h* and $h'$.

In the experimental analysis, the event shapes are computed from the hadron momenta in the current hemisphere, while the theoretical calculation uses the parton momenta. For the Born-level contribution to inclusive DIS, lepton-quark scattering, only the final state quark is produced in the current hemisphere, with thrust axis and current axis coinciding. Consequently, all event shape variables defined above become trivially zero. The first non-trivial contribution to the event shape distributions arises from two-parton final states: $eq \rightarrow eqg$ or $eg \rightarrow eq\bar{q}$, such that the leading-order (LO) perturbative contribution is $\mathcal{O}(\alpha _s)$. The event shape distributions are thus closely related to DIS two-jet production in the Breit frame.

In higher-multiplicity final states, it is possible that all partons scatter into the remnant hemisphere, leaving the current hemisphere empty. To ensure infrared safety of the observables, these events are not accepted by demanding that the total energy in the current hemisphere of an event exceeds some minimum value $\epsilon _{\mathrm {lim}}$

9 $$\begin{aligned} \sum _h E_h > \epsilon _{\mathrm {lim}}\,. \end{aligned}$$

Event shapes in deep inelastic scattering have been measured at HERA by the H1 [23] and ZEUS [24] experiments, based on the analysis of electron-proton scattering data taken at a centre-of-mass energy of $\sqrt{s}=319$ GeV (the H1 data set also contains a small fraction of data taken at $\sqrt{s} = 301$ GeV). The DIS kinematics in the process $e(k) + p(p) \rightarrow e(k') + X (p_X)$, with momentum transfer $q=k'-k$ is described by the variables $Q^2=-q^2$, $x=Q^2/(2q\cdot p)$ and $y=Q^2/(x s)$.

The H1 analysis [23] selects events with

10 $$\begin{aligned} 0.1<y<0.7, \quad 196~\text{ GeV }^2< Q^2 < 40000~\text{ GeV }^2, \end{aligned}$$

which are then classified into bins in $Q=\sqrt{Q^2}$, as listed in Table 1. For the event shape determination, $\epsilon _{\mathrm {lim}}= {Q}/{10}$ is used.

**Table 1:** Kinematic boundaries of the bins in *Q* in the H1 analysis [23]

Bin	$Q (\mathrm {GeV})$	
1	14	16
2	16	20
3	20	30
4	30	50
5	50	70
6	70	100
7	100	200

**Table 2:** Kinematic boundaries of the bins in $Q^2$ and *x* in the ZEUS analysis [24]

Bin	$Q^2 (\mathrm {GeV}^2)$		*x*	
1	80	160	0.0024	0.010
2	160	320	0.0024	0.010
3	320	640	0.01	0.05
4	640	1280	0.01	0.05
5	1280	2560	0.025	0.150
6	2560	5120	0.05	0.25
7	5120	10240	0.06	0.40
8	10240	20480	0.10	0.60

The ZEUS analysis [24] covers the kinematic range

11 $$\begin{aligned} 0.0024< x< 0.6\,, \quad 0.04< y< 0.9, \nonumber \\ 80~\text{ GeV }^2< Q^2 < 20480~\text{ GeV }^2, \end{aligned}$$

with events binned into in $(Q^2,x)$, described in Table 2. The energy cut in the current hemisphere used by ZEUS is $\epsilon _{\mathrm {lim}}= {Q}/{4}$.

Both experiments normalize the event shape distributions to the DIS cross section integrated over the kinematical bin under consideration, which is determined without applying the $\epsilon _{\mathrm {lim}}$ cut.

Both experiments performed measurements [23, 24] of the event shape distributions for $F=\tau _\gamma$, $\tau _T$, $\rho$, $B_\gamma$, *C*. In addition, they also measured the mean values $\langle F\rangle$ for these variables, supplemented in the ZEUS study by a measurement of the mean value $\langle B_T \rangle$ of the jet broadening with respect to the thrust axis. The measurements of the mean values are done for the same kinematical bins, Tables 1 and 2, as used for the distributions.

## QCD corrections to event shapes

The event shape variables defined above assume non-trivial values only for final states containing two or more partons. Consequently, the event shape distributions in DIS receive the same parton-level contributions as two-jet production in DIS. Higher-order QCD corrections to event shape distributions can thus be obtained from the corresponding calculation for di-jet production by replacing the jet reconstruction algorithm by computations of the event shape variables.

We calculate the differential distributions and mean values for the DIS event shapes with the parton-level Monte Carlo event generator $\hbox {NNLO}{{\textsc {jet}}}$, by extending the existing calculation of NNLO corrections to di-jet production in DIS [26, 27]. It combines the contributions from four-parton production at tree-level [31–33], three-parton production at one loop [34–37] and two-parton production at two loops [38–41], using the antenna subtraction method [42–44] to isolate infrared singular terms from the different contributions, which are then combined to yield numerically finite predictions for arbitrary infrared-safe observables constructed from the parton momenta. Besides for di-jet production at NNLO, the same ingredients and setup have been used previously in the computation of N$^3$LO corrections to single jet production in DIS [45], in extractions of the strong coupling constant from DIS jet data [46, 47], and in studies of diffractive di-jet production [48]. The calculations have also been extended to jet production in charged current DIS [49, 50] at the same perturbative orders.

We compute the event shapes for electron-proton collisions with $\sqrt{s}=319$ GeV, using the NNPDF3.1 parton distributions with $\alpha _s(M_Z)=0.118$ and for $N_F=5$ massless quark flavours. Central renormalization and factorization scales are fixed to $\mu _F=\mu _R=Q$, and theory uncertainties are estimated by the envelope of varying these scales independently by a factor two up and down, avoiding the pairings of variations in opposite directions (seven-point scale variation). Event selection cuts on the lepton variables and on $\sum _h E_h$ are applied according to the H1 [23] and ZEUS [24] analyses, and events are then classified into the different kinematical bins of Tables 1 and 2. The total hadronic DIS cross section for each kinematical bin (required for the normalization of the event shape distributions and mean values) is obtained to NNLO from $\hbox {NNLO}{{\textsc {jet}}}$, based on the one-jet calculation to this order [45]. Central renormalization and factorization scales are used for the normalization.

### Event shape distributions

The event shape distributions are computed as histograms in the event shape variables. We use a considerably finer bin resolution than in the experimental analyses [23, 24], which will subsequently allow us to apply hadronization corrections that result in a dynamical shift of the event shape variables. The histograms are defined in terms of variable ranges and number of equal-sized bins:12The fixed-order calculation for an event shape *F* diverges in the limit $F\rightarrow 0$, where all-orders resummation of large $\log (F)$-terms is required. In this limit, the fixed-order expressions become meaningless, and we accordingly apply cuts on the minimum values of each shape variable, which set the first few bins of the distributions to zero:13These cuts are typically within the first bin of the experimental analysis, which should anyhow be discarded in the comparison of fixed-order theory and experimental data.

**Fig. 1:**
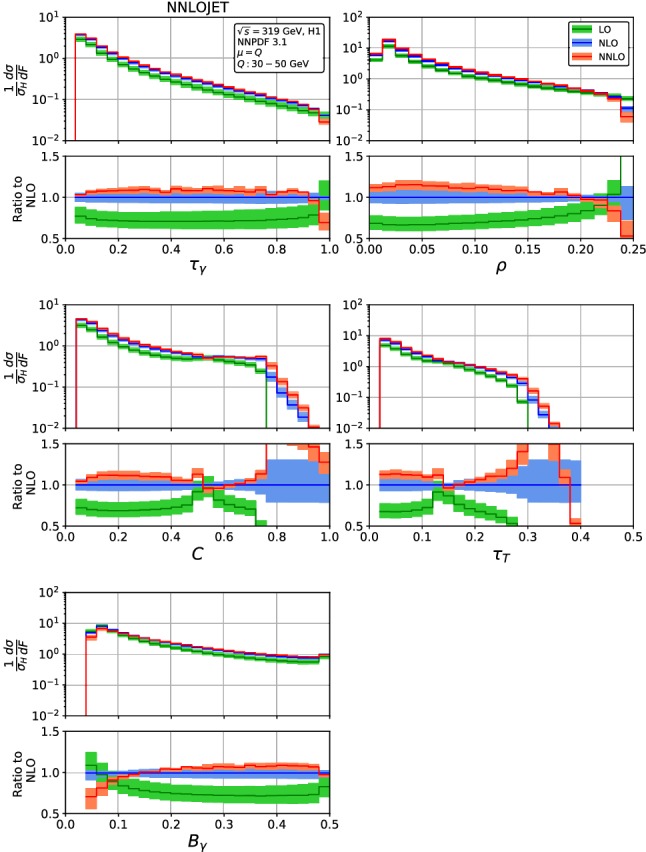
Fixed-order predictions for the event shape distribution for H1 kinematics [23] in *Q* = 30–50 GeV bin: LO (green), NLO (blue) and NNLO (red), for H1 kinematics [23]. The lower frames display the ratio to the NLO predictions for the central scale $\mu ^2=Q^2$

Figure 1 displays the fixed-order predictions (re-binned from the initial histograms by combining four adjacent bins each) for the H1 kinematics in the $Q=30-50$ GeV bin. Since the qualitative behaviour of the higher-order corrections to the distributions is similar for all kinematical bins, we show the fixed-order distributions without power corrections only for one representative bin. The quantitative size of the corrections and of their uncertainties decreases with increasing *Q*, mainly due to the decrease in the running coupling constant $\alpha _s$.

In general, we observe that the NNLO corrections in the bulk of all distributions are typically positive (up to + 20%), often displaying only a marginal or no overlap of the uncertainty bands at NLO and NNLO. The scale uncertainty decreases from NLO ($\sim$10%) to NNLO ($\sim$5%). Even in the bulk, the higher-order corrections are not uniform between the distributions, each displaying a non-trivial shape in the NNLO/NLO ratio.

Towards the kinematical edges $F\rightarrow 0$ and $F\rightarrow F_{\mathrm{max}}$, the higher-order corrections behave differently for each distribution, often displaying large effects well beyond the scale uncertainty estimates. For $F\rightarrow F_{\mathrm{max}}$, these features are caused by two different but related issues. For some of the shape variables, $F_{\mathrm{max}}$ can not yet be realised in the Born process, owing to its low multiplicity. This is the case for the *C*-parameter which has a Born-level upper limit of 3 / 4 and for $\tau _T$ with an upper limit of 0.293. Higher order real radiation corrections allow to attain larger values of *F*, thereby resulting in a kinematical mismatch between real and virtual contributions (Sudakov shoulder, [51]), which (although finite) produces large perturbative corrections in the vicinity of the Born-level kinematical limit.

In the case of DIS event shape variables, the kinematical constraints of the Born process produce further structures that narrow down the dimensionality of the final state phase space for specific values of different variables. These ridges in the multi-dimensional phase space were investigated in detail in [21] and produce kinks and spikes in the one-dimensional event shape distributions. These are sometimes already present at leading order, and go along with large and unstable higher order corrections in the immediate vicinity of the exceptional points, which are visible in particular in the distributions in *C* and $\tau _T$ in Fig. 1. These features are typically localised in small patches of the phase space. For sufficiently large bin sizes, their impact is diluted to an invisible level. High-resolution measurements of event shape distributions, for example at a future electron-ion collider [52] or at the LHeC [53] will be able to resolve these features, thereby potentially necessitating resummation of large corrections associated with them.

At low values of *F*, the fixed-order predictions contain logarithmic terms $\log F$ at each order in perturbation theory, which spoil the convergence of the fixed-order perturbative expansion. In Fig. 1, the onset of these effects is visible in particular in the $B_\gamma$ distribution, while its onset takes place only at lower values of *F* in all other distributions. A description of the event shape distributions over the full kinematical range, and extending towards lower values of *F* than probed by currently available measurements [23, 24] will need to include the resummation of these $\log F$ terms, which is currently known to next-to-leading logarithmic level [19–22] for all distributions.

### Mean values

**Fig. 2:**
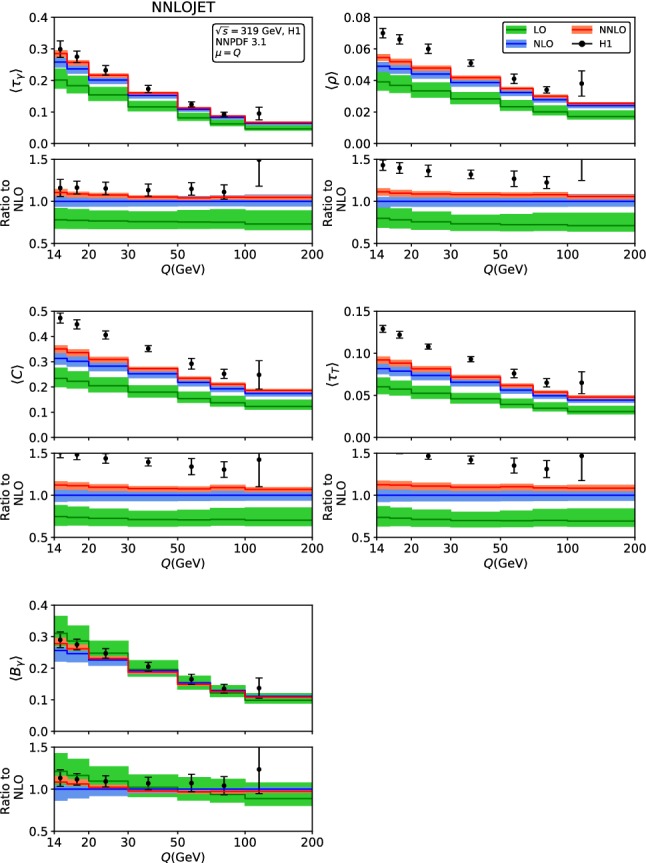
Fixed-order predictions for the mean value of the event shapes at LO (green), NLO (blue) and NNLO (red) compared to H1 data [23]

**Fig. 3:**
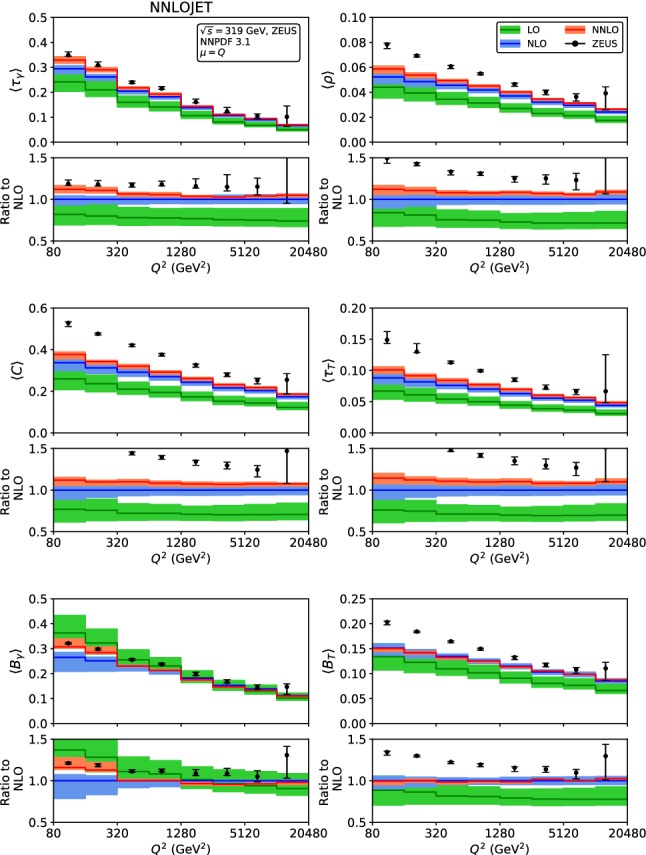
Fixed-order predictions for the mean value of the event shapes at LO (green), NLO (blue) and NNLO (red) compared to ZEUS data [24]

The mean values of the different event shapes variables are computed using $\hbox {NNLO}{{\textsc {jet}}}$by weighting each event with the reconstructed value of the event shape variable under consideration. The phase space integrations are performed by imposing only a very low technical cut-off of $F_{\mathrm{min}}=10^{-4}$ on the event shape variables, since the weighting with the shape variable regulates the divergent behaviour of the integrals for $F\rightarrow 0$, rendering the mean value integrals finite. The numerical stability of the mean value integrals has been checked with an even lower technical cut-off of $F_{\mathrm{min}}=10^{-5}$, from which we conclude that $F_{\mathrm{min}}=10^{-4}$ is already sufficient for a stable result. The mean values are also normalized to the inclusive hadronic cross sections.

The fixed-order predictions for the mean values are displayed in Figs. 2 and 3, for the H1 [23]and ZEUS [24] kinematics. With the exception of the broadenings $\langle B_\gamma \rangle$ and $\langle B_T\rangle$, the NNLO corrections to the mean values are positive for all event shapes, and decrease in magnitude with increasing $Q^2$. The NNLO predictions are often at the upper boundary of the NLO theory uncertainty band, for the lowest $Q^2$ bins they are even outside the NLO band. For the broadenings, the NNLO corrections to $\langle B_\gamma \rangle$ are positive at low $Q^2$, and become negative at large $Q^2$, to $\langle B_T\rangle$ they display the opposite behaviour, and are smaller in absolute magnitude. For all mean values, inclusion of the NNLO corrections leads to a reduction of the scale uncertainty compared to NLO, which is most pronounced for the broadenings, whereas being more modest for the other shape variables. For large values of $Q^2>2500$ GeV$^2$, the NNLO theory uncertainty is limited to below 5%.

Comparing the fixed-order predictions to the measurements of the mean values from H1 and ZEUS, we observe that the data are considerably above the theory predictions throughout all shape variables and for all values of $Q^2$, although the discrepancy is most pronounced at low $Q^2$. This behaviour indicates the relevance of power corrections from hadronization effects, which can have large effects on the mean values [28–30].

## Hadronization effects

In the previous section, we computed higher-order corrections to the DIS event shape distributions and mean values at parton level. To compare these predictions with hadron-level data requires accounting for the impact of the parton–hadron transition, which is a non-perturbative process. Consequently, these hadronization effects cannot be computed in perturbation theory, but require a non-perturbative model description. The hadronization corrections are expected to be suppressed by positive powers of $\Lambda /Q$, such that their relative numerical impact is decreasing with increasing *Q*. In the following, we employ the dispersive model [28] to estimate the leading power corrections at order $(\Lambda /Q)$ to event shape distributions. This model has been worked out in detail for the DIS event shapes in Ref. [29], and its implications are briefly summarized in the following.

In the dispersive model, an effective coupling $\alpha _{\mathrm {eff}}$ is introduced at low scales, which is matched to the running QCD coupling $\alpha _s(\mu )$ at a scale $\mu _I = 2$ GeV. This gives a constant $\alpha _0$ which is defined as the first moment of the effective coupling below the scale $\mu _I$,

14 $$\begin{aligned} \alpha _0(\mu _I) = \frac{1}{\mu _I} \int _0^{\mu _I} \mathrm {d}\mu \, \alpha _{\mathrm {eff}}(\mu )\,. \end{aligned}$$

The power corrections are suppressed by powers of 1 / *Q*, and result in a shift of the perturbative differential distribution

15 $$\begin{aligned} \frac{\mathrm {d}\sigma ^{\mathrm {hadron}}(F)}{\mathrm {d}F} = \frac{\mathrm {d}\sigma ^{\mathrm {parton}}(F - a_F P)}{\mathrm {d}F}, \end{aligned}$$

where the power correction *P* is universal for all the event shape variables. The perturbative ingredients to the dispersive model are the running of the coupling constant and the relation between the $\overline{\mathrm{MS}}$-coupling and the effective coupling, whose definition [54] absorbs universal correction terms from the cusp anomalous dimension. It can be expanded in $\alpha _s(Q)$, and its expression up to NNLO is given by [55, 56]:

16 $$\begin{aligned} P= & {} \frac{8 C_F}{\pi ^2} \mathcal {M} \frac{\mu _I}{Q} \bigg \{ \alpha _0(\mu _I) - \alpha _s(Q) \nonumber \\&- \frac{\beta _0}{2\pi } \left( \log \frac{Q}{\mu _I} + \frac{K}{\beta _0} + 1 \right) \alpha _s^2(Q) \nonumber \\&- \bigg [ \frac{\beta _1}{2} \left( \log \frac{Q}{\mu _I} + \frac{2 L}{\beta _1} + 1 \right) \nonumber \\&+ 2\beta _0^2 \left( \log \frac{Q}{\mu _I} + \frac{K}{\beta _0} + 1 \right) \nonumber \\&+ \beta _0^2 \log \frac{Q}{\mu _I} \left( \log \frac{Q}{\mu _I} + \frac{2 K}{\beta _0} \right) \bigg ] \frac{\alpha _s^3(Q)}{4\pi ^2} \bigg \}, \end{aligned}$$

with $\mathcal {M} = 1.49$ a constant normalization factor (Milan factor [30, 57]) accounting for higher-order contributions. In our numerical results, we use $\alpha _0(\mu _I) = 0.5$ at $\mu _I=2$ GeV, which has been estimated from fits to event shape moments in DIS [23, 24, 58] and $e^+e^-$ annihilation [56]. The beta-function coefficient and cusp anomalous dimension [59] in the above expression are:

17 $$\begin{aligned} \beta _0= & {} \frac{11}{3} C_A - \frac{4}{3} T_F N_F, \nonumber \\ \beta _1= & {} \frac{34}{3} C_A^2 - \frac{20}{3} C_A T_F N_F - 4 C_F T_F N_F, \nonumber \\ K= & {} \left( \frac{67}{18} - \frac{\pi ^2}{6}\right) C_A - \frac{10 T_F N_F}{9}, \nonumber \\ L= & {} C_A^2 \left( \frac{245}{24} - \frac{67}{9} \frac{\pi ^2}{6} + \frac{11}{6} \zeta _3 + \frac{11}{5} \bigg ( \frac{\pi ^2}{6} \bigg )^2 \right) \nonumber \\&+ C_A N_F \left( -\frac{209}{108} + \frac{10}{9} \frac{\pi ^2}{6} - \frac{7}{3} \zeta _3 \right) \nonumber \\&+ C_F N_F \left( -\frac{55}{24} + 2 \zeta _3 \right) + N_F^2 \left( -\frac{1}{27} \right) , \end{aligned}$$

with $C_A=3$, $C_F=4/3$, $T_F=1/2$. The coefficients $a_F$ depend on the event shape variable, they were computed in [29] and are tabulated in [58]. Their values are repeated here:

18 $$\begin{aligned} a_{\tau _\gamma }= & {} 1\,, \quad a_{\tau _T} = 1\,, \quad a_\rho = \frac{1}{2}\,, \nonumber \\ a_{B_\gamma }= & {} \frac{1}{2} a'_B\,, \quad a_{B_T} = \frac{1}{2} a'_B\,, \quad a_C = \frac{3}{2} \pi \,, \end{aligned}$$

where the shift of the jet broadening has an additional enhancement [60] given by

19 $$\begin{aligned} a'_B = \frac{\pi }{2 \sqrt{2C_F \alpha _s (1+\frac{K}{2\pi } \alpha _s)}} + \frac{3}{4} - \frac{\beta _0}{12C_F} + \eta _0, \end{aligned}$$

with $\alpha _s$ evaluated at the scale $\mu =e^{-\frac{3}{4}} \mu _R$ and $\eta _0 = -0.614$. For this analysis $a'_B$ varies from 1.6 to 2.3.

The dispersive model is based on an analytic treatment of hadronization effects on a two-parton correlator [28], which corresponds to the mean value integral of each event shape. The effect of the dispersive power correction on the mean values is additive:

20 $$\begin{aligned} \langle F \rangle = \langle F \rangle ^{\mathrm{pert.}} + a_F\, P, \end{aligned}$$

where $\langle F \rangle ^{\mathrm{pert.}}$ is the mean value obtained in fixed-order perturbation theory, described in Sect. 3.2 above.

**Fig. 4:**
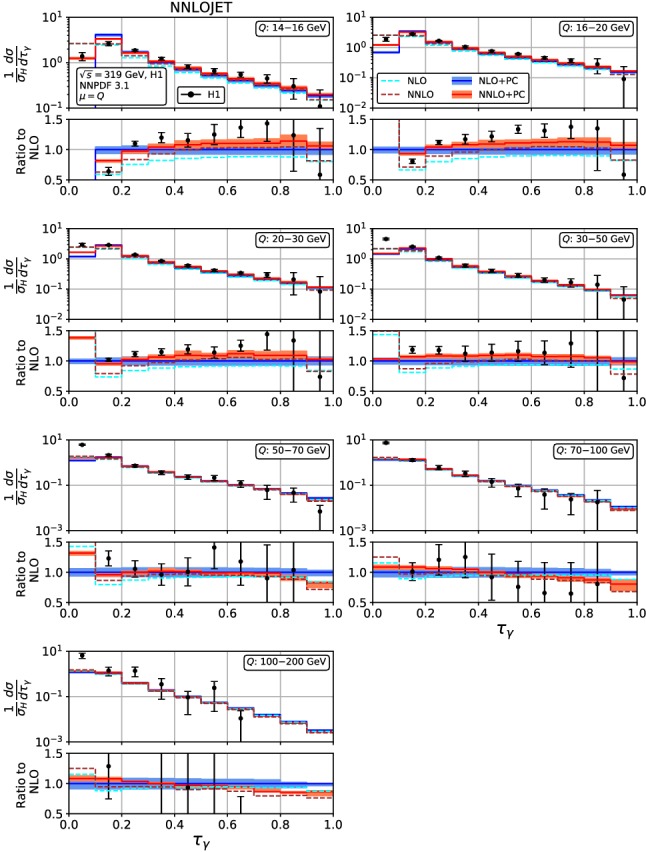
Event shape distribution for thrust with respect to boson axis: $\tau _\gamma$ fixed-order predictions at NLO (dashed cyan), NNLO (dashed brown), and corrected for hadronization effects at NLO (blue) and NNLO (red), compared to H1 data [23]. The lower frames display the ratio to the NLO prediction for the central scale $\mu ^2=Q^2$

**Fig. 5:**
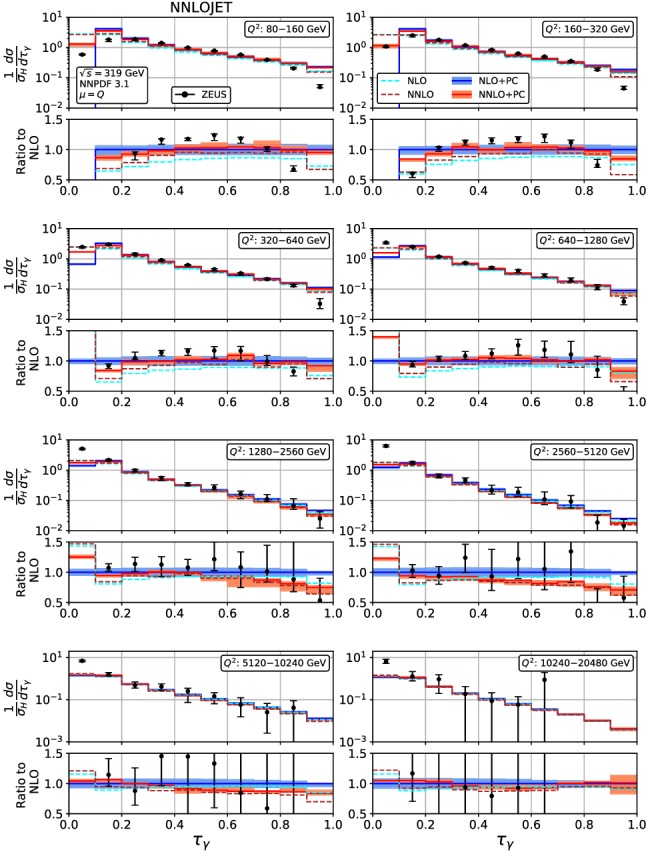
Event shape distribution for thrust with respect to boson axis: $\tau _\gamma$ fixed-order predictions at NLO (dashed cyan) and NNLO (dashed brown), and corrected for hadronization effects at NLO (blue) and NNLO (red), compared to ZEUS data [24]. The lower frames display the ratio to the NLO prediction for the central scale $\mu ^2=Q^2$

When applied to differential event shape distributions, the power correction *P* in the shift (15) can in principle depend on the numerical value of *F*. Using a constant shift *P* for the full distribution amounts to an approximation, which may be overcome by an improved treatment of the non-perturbative corrections.

In combining the fixed-order predictions derived in the previous section with the power corrections, we truncate the factor *P* in (16) to $\alpha _s^2(Q)$ for NLO, and to $\alpha _s^3(Q)$ for NNLO. Inclusion of the $\alpha _s^3(Q)$ terms leads to a substantial reduction of *P*, with $P_{\mathrm{NNLO}} \approx 0.60 \, P_{\mathrm{NLO}}$ at $Q=15$ GeV and $P_{\mathrm{NNLO}} \approx 0.75 \, P_{\mathrm{NLO}}$ at $Q=100$ GeV.

## Results

With the inclusion of power corrections, the fixed-order parton-level predictions can now be compared to hadron-level data from the HERA experiments on event shape distributions and mean values.

**Fig. 6:**
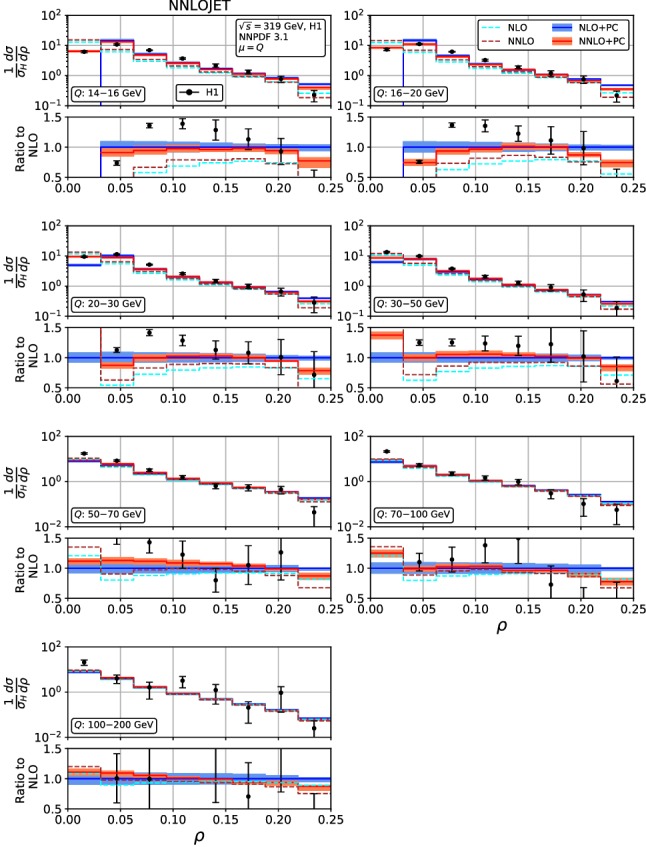
Event shape distribution for jet mass: $\rho$ fixed-order predictions at NLO (dashed cyan), NNLO (dashed brown), and corrected for hadronization effects at NLO (blue) and NNLO (red), compared to H1 data [23]. The lower frames display the ratio to the NLO prediction for the central scale $\mu ^2=Q^2$

**Fig. 7:**
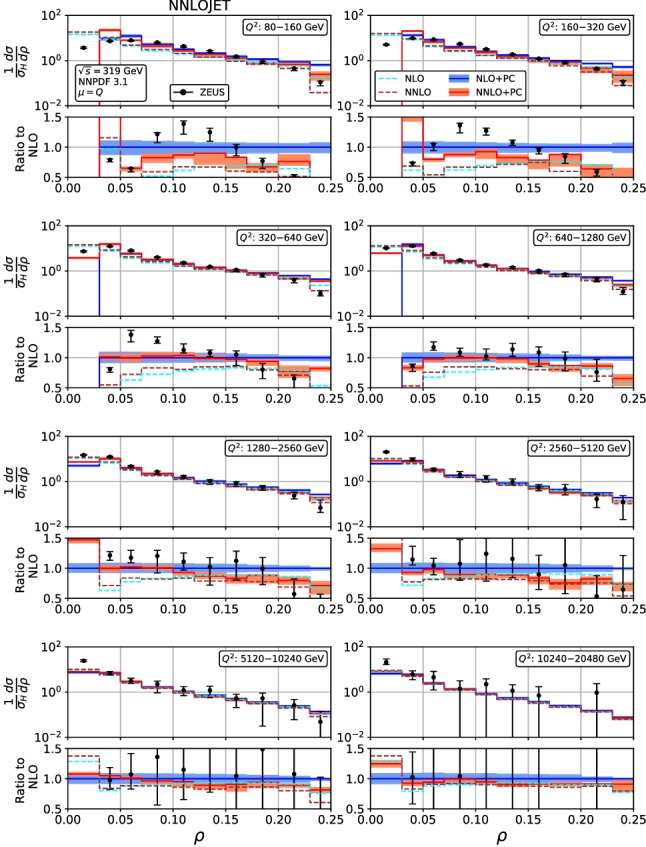
Event shape distribution for jet mass: $\rho$ fixed-order predictions at NLO (dashed cyan) and NNLO (dashed brown), and corrected for hadronization effects at NLO (blue) and NNLO (red), compared to ZEUS data [24]. The lower frames display the ratio to the NLO prediction for the central scale $\mu ^2=Q^2$

### Event shape distributions

**Fig. 8:**
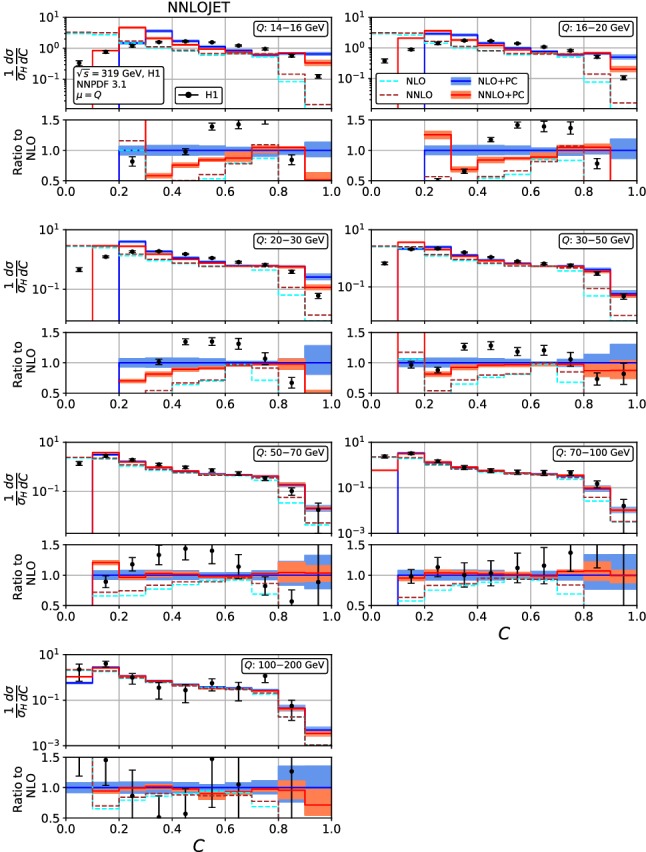
Event shape distribution for *C*-parameter: *C* fixed-order predictions at NLO (dashed cyan), NNLO (dashed brown), and corrected for hadronization effects at NLO (blue) and NNLO (red), compared to H1 data [23]. The lower frames display the ratio to the NLO prediction for the central scale $\mu ^2=Q^2$

**Fig. 9:**
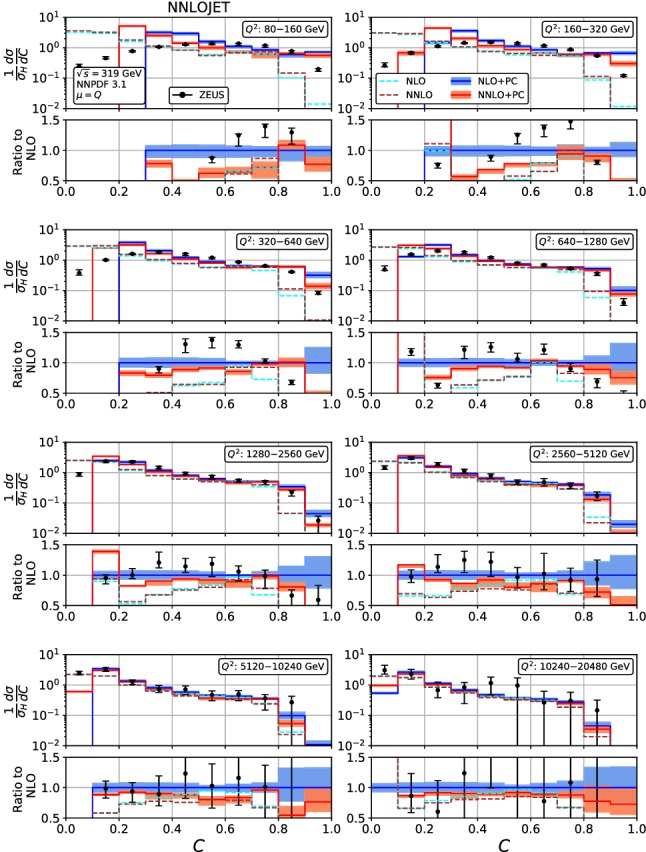
Event shape distribution for *C*-parameter: *C* fixed-order predictions at NLO (dashed cyan) and NNLO (dashed brown), and corrected for hadronization effects at NLO (blue) and NNLO (red), compared to ZEUS data [24]. The lower frames display the ratio to the NLO prediction for the central scale $\mu ^2=Q^2$

**Fig. 10:**
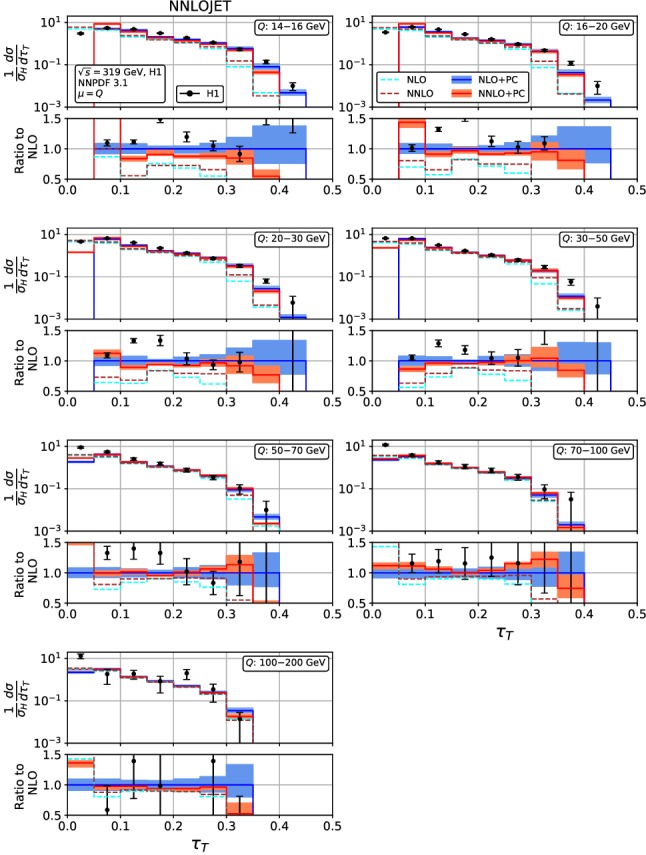
Event shape distribution for thrust with respect to thrust axis: $\tau _T$ fixed-order predictions at NLO (dashed cyan), NNLO (dashed brown), and corrected for hadronization effects at NLO (blue) and NNLO (red), compared to H1 data [23]. The lower frames display the ratio to the NLO prediction for the central scale $\mu ^2=Q^2$

**Fig. 11:**
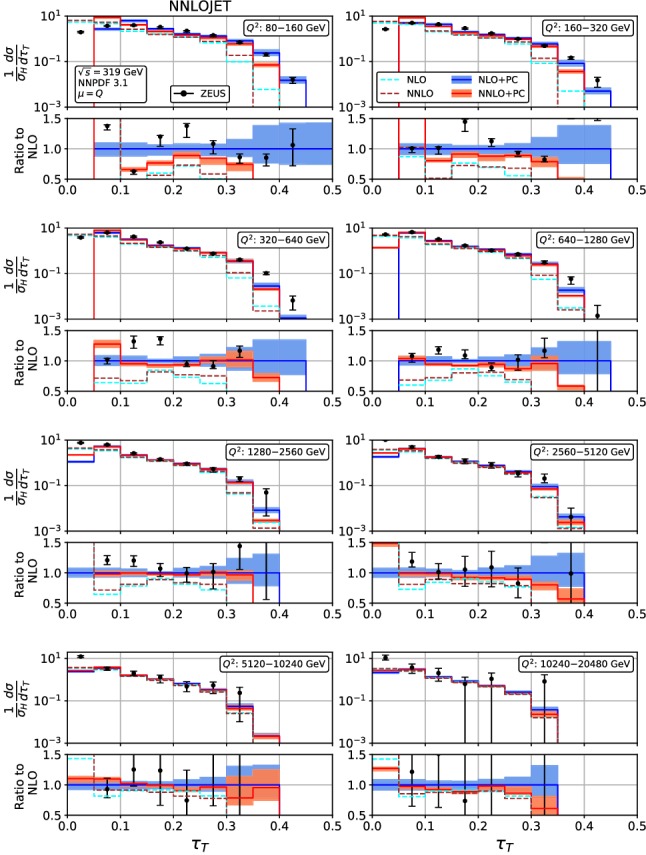
Event shape distribution for thrust with respect to thrust axis: $\tau _T$ fixed-order predictions at NLO (dashed cyan) and NNLO (dashed brown), and corrected for hadronization effects at NLO (blue) and NNLO (red), compared to ZEUS data [24]. The lower frames display the ratio to the NLO prediction for the central scale $\mu ^2=Q^2$

**Fig. 12:**
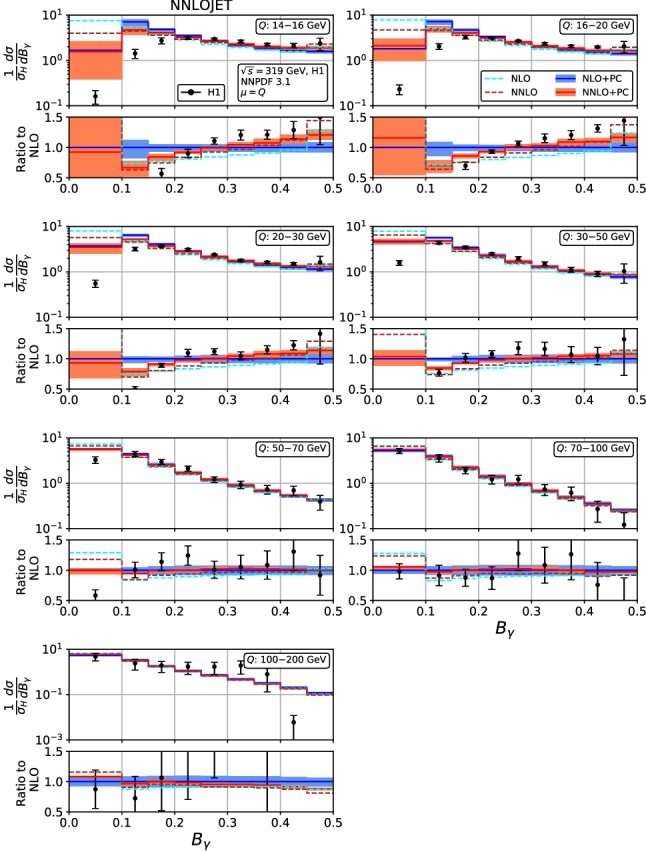
Event shape distribution for jet broadening with respect to thrust axis: $B_\gamma$ fixed-order predictions at NLO (dashed cyan), NNLO (dashed brown), and corrected for hadronization effects at NLO (blue) and NNLO (red), compared to H1 data [23]. The lower frames display the ratio to the NLO prediction for the central scale $\mu ^2=Q^2$

**Fig. 13:**
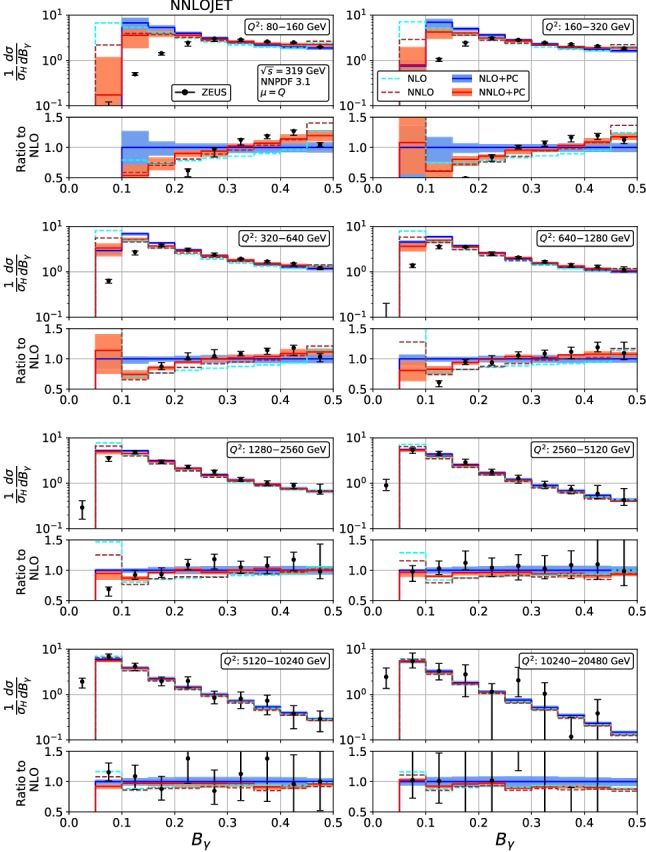
Event shape distribution for jet broadening with respect to thrust axis: $B_\gamma$ fixed-order predictions at NLO (dashed cyan) and NNLO (dashed brown), and corrected for hadronization effects at NLO (blue) and NNLO (red), compared to ZEUS data [24]. The lower frames display the ratio to the NLO prediction for the central scale $\mu ^2=Q^2$

Figures 4, 5, 6, 7, 8, 9, 10, 11, 12 and 13 display the theory predictions obtained by combining the fixed-order predictions up to NNLO with power corrections computed using the dispersive model as described in the previous section to the experimental data from H1 [23] and ZEUS [24]. To illustrate the magnitude of the power corrections, the uncorrected fixed-order predictions for central scales $\mu =Q$ are indicated by blue lines at NLO and brown lines at NNLO. The shift (15) is applied on the high-resolution histograms (12) which were computed with a lower cut-off affecting their first bin (where all-order resummation of large logarithmic corrections [19–22] is required to obtain a finite prediction). The shifted high-resolution histograms are then combined to the number of bins used in the experimental measurements:21Owing to the interplay of the lower cut-off on the distributions and the power correction shift, the prediction for the left-most non-vanishing bin of each distribution is unreliable, and should not be taken into account when comparing the experimental data with the theory predictions. A prediction for $F\rightarrow 0$ will have to include resummation in order to become meaningful. This limitation should be kept in mind in the following comparisons.

For the thrust distribution $\tau _\gamma$, Figs. 4 and 5, we observe that the NNLO corrections at low and moderate values of $Q^2$ are leading to an increase of the distribution in the bulk, and a slight decrease at high and low $\tau _\gamma$. At the highest values of $Q^2$, the NNLO corrections become very small and negative even in the bulk. Overall, the NNLO corrections improve the description of the data. Compared to NLO, inclusion of the NNLO correction leads to a reduction of the scale uncertainty. This reduction is only moderate at the lowest values of $Q^2$, where the NNLO scale uncertainty remains at the 6% level. At higher $Q^2$, the reduction of scale uncertainty at NNLO is more pronounced, leading to predictions with residual uncertainty below 4%. These uncertainties should be compared to the experimental errors. The ZEUS data [24] are slightly more precise than the H1 data [23], and also reach to lower values of $Q^2$. In the low-$Q^2$ bins, the NNLO scale uncertainty remains larger than the experimental errors, as was also observed [61] for jet production in DIS at low $Q^2$. For moderate and high values of $Q^2$, the scale uncertainty is now well below the experimental errors, thereby allowing for the use of the event shape distributions in precision QCD studies.

A similar pattern is also observed in the jet mass distribution, Figs. 6 and 7: positive NNLO corrections in the bulk at moderate $Q^2$, which turn negative when going to large values of $\rho$ or to large $Q^2$. At the lowest values of $Q^2$, the NNLO corrections are negative throughout the distribution (despite positive corrections at parton-level) due to the reduced size of the power correction at NNLO. The NNLO corrections lead to an improved description of the shape of the experimental data. This improvement is particularly visible at large $\rho$ for all values of $Q^2$, where negative NNLO contributions lead to a considerably better description of the kinematical shape of the data. Overall, the agreement between data and theory is however somewhat worse for $\rho$ than it was for $\tau _\gamma$. The NNLO scale uncertainties also follow a similar pattern as for $\tau _\gamma$: compared to NLO only a modest reduction at low $Q^2$ and a substantial reduction to the level of a few per cent at high $Q^2$. Again, the experimental errors are larger than the scale uncertainty for moderate and high $Q^2$, thus enabling precision QCD studies.

In the *C*-parameter, Figs. 8 and 9, we must distinguish the region below and above the Sudakov shoulder, which is located at $C=0.75$ in the perturbative parton-level expression. The dispersive power corrections shift the location of this shoulder to higher values of *C*. This shift is largest at low $Q^2$, and decreases in magnitude towards higher $Q^2$. The region above the Sudakov shoulder is kinematically forbidden at LO, and receives contributions only from NLO onwards. Already for values of *C* below the Sudakov shoulder, the pattern of NNLO corrections is more intricate than what was observed in $\tau _\gamma$ and $\rho$. The observed structure is due to presence of a kinematical ridge [21] in the perturbative expressions at $C\approx 0.515$, which destabilizes the perturbative convergence of the distribution in its vicinity, clearly visible in the high-resolution *C*-parameter distribution, Fig. 1. The perturbative predictions for the *C*-parameter distributions become quite precise at NNLO, with scale uncertainties of typically less than 8% below the Sudakov shoulder and away from the kinematical ridge. They are however affected by large hadronization corrections, which shift the whole distribution by more than two bins in *C* at low $Q^2$. Compared to all other event shape distributions, these power corrections are particularly large in the *C*-parameter distributions, see (18). For the lower values of $Q^2$, we also observe that the shape of the distribution is poorly described. For medium and large values of $Q^2$, the power corrections are much smaller, NNLO corrections are relatively small and uniform, and a satisfactory description of the experimental data is observed.

The thrust distribution with respect to the thrust axis $\tau _T$, Figs. 10 and 11, displays a similar pattern, with non-trivial structures in its perturbative expressions around the Sudakov shoulder at $\tau _T=0.293$ and the kinematical ridge around $\tau _T\approx 0.13$, which are both nicely visible in the $\tau _T$-distribution at high resolution, Fig. 1. These exceptional points are shifted to larger values of $\tau _T$ by the power corrections. The NNLO corrections are negative and small throughout almost the whole distribution for all values of $Q^2$, except in the bin with the highest $\tau _T$, which is already well above the Sudakov shoulder, and where the cross section is already very small. As for $\tau _\gamma$, we note that the smallness of the NNLO effect is mainly due to a cancellation between positive parton-level corrections and a decrease in the power corrections. The corrections are typically within the NLO scale uncertainty band. The overall agreement between experimental data and theory predictions is satisfactory only at higher $Q^2$. Substantial discrepancies at low $Q^2$ are observed especially in the vicinity of the kinematical ridge, where the theory predictions are systematically and considerably below the data. This behaviour may be indicative of the need of an re-consideration of the perturbative expansion and of the hadronization corrections in regions around the kinematical ridges.

Finally, for the jet broadening with respect to the boson axis $B_\gamma$, Figs. 12 and 13, the NNLO corrections assume a non-trivial shape, changing from negative at small $B_\gamma$ to positive at large $B_\gamma$, thereby leading to a considerably better description of the data. In these distributions, the onset of large logarithmic terms at low $B_\gamma$ is well visible, indicating the need for their resummation. The NNLO corrections lead to a considerable reduction of the scale uncertainty in the bulk of the distributions, which is more pronounced at low $Q^2$ than in most other event shape distributions. The NNLO scale uncertainty ranges from 8% at low $Q^2$ to 3% at high $Q^2$, which is comparable to or below the experimental uncertainties throughout.

Across the different event shape distributions, several common features are observed. The NNLO corrections are typically moderate, and fall usually within the NLO scale uncertainty bands. This is particularly remarkable since the NLO corrections were typically large (often comparable in size to the LO predictions), and well outside the LO scale uncertainty bands. The numerical smallness of the NNLO effect is often due to a partial cancellation between the parton-level correction and modification of the power corrections at this order. Except in the low-*F* region, where large logarithmic corrections require an all-order resummation, and in the vicinity of Sudakov shoulders and kinematical ridges, we observe the onset of a good convergence of the perturbative fixed-order expansion. The corrections at low values of $Q^2$ are inevitably larger (due to the larger expansion parameter), which also translates in a sizeable scale uncertainty remaining at NNLO of about 10%. At larger values of $Q^2$, this scale uncertainty improves considerably to the typically 4% or below, clearly highlighting the potential of precision QCD studies with event shapes based on existing HERA data [23, 24], or with much larger data sets for hadronic final states that could be obtained at a future electron-ion collider [52] or at the LHeC [53]. The non-perturbative power corrections that we obtained in the dispersive model induce large shifts in some of the distributions, especially at low $Q^2$, where the statistical quality of the data is largest. Moreover, their application to the distributions in the form of a constant shift is only an approximation, which should be revisited carefully as soon as more precise data are becoming available.

### Mean values

**Fig. 14:**
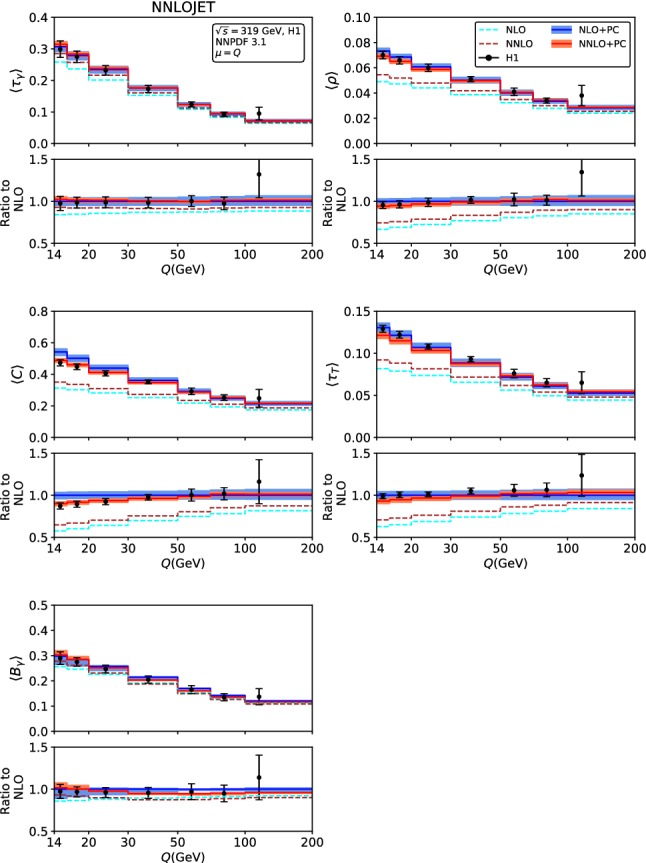
Mean value of the event shapes at NLO (blue) and NNLO (red) including power corrections, compared to H1 data [23]. The fixed-order NLO and NNLO predictions (dashed cyan and brown lines) are included to illustrate the magnitude of the power corrections

**Fig. 15:**
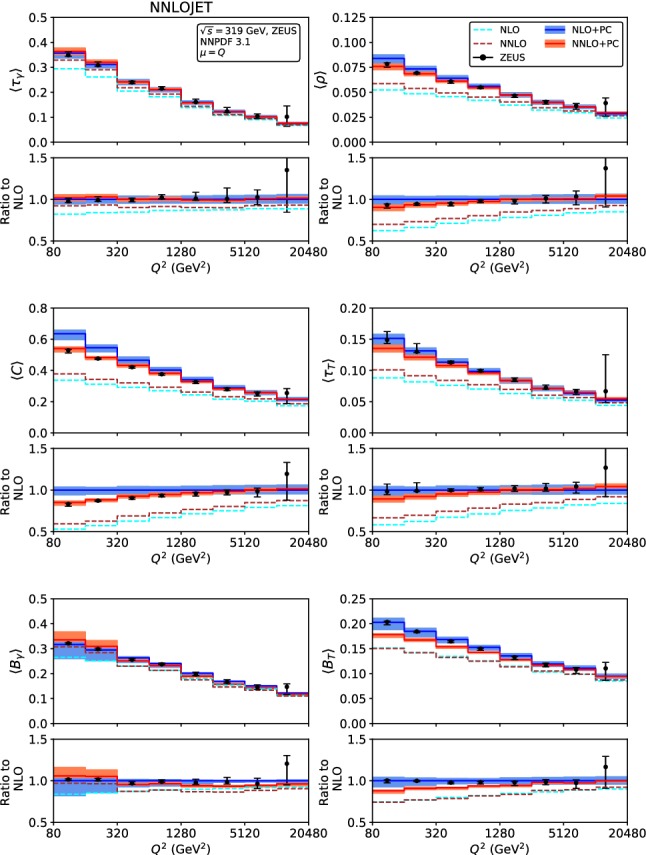
Mean value of the event shapes at NLO (blue) and NNLO (red) including power corrections, compared to ZEUS data [24]. The fixed-order NLO and NNLO predictions (dashed cyan and brown lines) are included to illustrate the magnitude of the power corrections

The power corrections to the mean values result in an additive shift of the perturbative predictions, see (20). As for the event shape distributions, we truncate *P* in (16) to order $\alpha _s^2(Q)$ for the power corrections to the NLO fixed order predictions and to $\alpha _s^3(Q)$ for power corrections applied to the NNLO predictions. Applying this shift to the perturbative results of Sect. 3.2, we obtain hadron-level predictions for the mean values, which are compared to the data from H1 [23] and ZEUS [24] in Figs. 14 and 15. The fixed-order predictions for central scales $\mu =Q$ are indicated by blue lines at NLO and brown lines at NNLO, showing that the power corrections are sizeable for all mean values. Their inclusion eliminates the tension between data and purely perturbative results seen in Figs. 2 and 3 above.

Comparing the mean values with and without power corrections, we observe that the large positive NNLO corrections at low $Q^2$ that are seen in Figs. 2 and 3 are more than compensated by the decrease in the numerical magnitude of the power correction in going from NLO to NNLO in *P*. The combined effect of the NNLO contributions to the fixed order predictions and the power corrections is typically a small reduction of the mean values at low $Q^2$, thereby leading to an improved description of the H1 and ZEUS data. At larger $Q^2$, this combined effect results in a very small change of the predictions from NLO to NNLO, which comes with a substantial reduction of the perturbative scale uncertainty, which is almost halved.

Both the H1 [23] and ZEUS [24] studies used their measurements of event shape distributions for a simultaneous fit of the QCD coupling constant $\alpha _s(M_Z)$ and the effective coupling $\alpha _0$ that appears in the power correction, performed using NLO fixed-order results. While ZEUS lists only the results obtained for the individual event shape variables (displaying a substantial scatter), H1 also performed a combined fit, resulting in $$\alpha _s(M_Z) = 0.1198\pm 0.0012 \mathrm{(exp)} {\begin{array}{c} +0.0056 \\ -0.0043 \end{array}} \mathrm{(th)}$$ and $$\alpha _0 = 0.476\pm 0.008 \mathrm{(exp)} {\begin{array}{c} +0.018 \\ -0.059 \end{array}} \mathrm{(th)}$$. Especially the theory error on $\alpha _s(M_Z)$ is largely dominated by the NLO scale uncertainty.

A similar NNLO study has been performed previously on event shape moments in $e^+e^-$ annihilation [56], resulting in decreased scatter between different shape variables, and in lower theory uncertainties. Also, a slight increase in the fitted central value of $\alpha _0$ from NLO to NNLO was observed in $e^+e^-$, resulting in an NNLO value of $\alpha _0 =0.5132\pm 0.0115\text{(exp) }\pm 0.0381\text{(th) }$. Our predictions in Figs. 14 and 15 use $\alpha _0 =0.5$ throughout. They result in a very good description of the data at NNLO, and we notice that the NLO curves could be brought into better agreement with the data by a slight lowering of $\alpha _0$, towards its H1 fit value.

With the newly derived NNLO corrections, the combined fit of $\alpha _s(M_Z)$ and $\alpha _0$ to event shape distributions and their mean values can now be repeated fully consistently to NNLO accuracy. It can be anticipated that the main effect of the NNLO corrections will be in a reduction of the theory-induced uncertainty on the extracted value of $\alpha _s(M_Z)$, which was found to be about 5% in the NLO-based study by H1 [23]. This reanalysis of the experimental data will require our fixed-order results to be re-cast into convolution grids [47] that enable an efficient re-evaluation for multiple parameter values and parton distributions, which is beyond the scope of the present paper.

## Conclusions

In this paper, we computed the NNLO QCD corrections to event shape distributions and their mean values in deep inelastic lepton–proton scattering. Our calculation was performed in the $\hbox {NNLO}{{\textsc {jet}}}$framework, and is largely based on the NNLO corrections [26, 27] to di-jet production in DIS. The NNLO corrections to the distributions are not uniform, although some general trends are observed: positive corrections in the bulk of the distributions at low and medium $Q^2$, negative corrections in the bulk at high $Q^2$ and at the upper kinematical boundaries of the shape variables for all $Q^2$. Several perturbative instabilities due to Sudakov shoulders [51] or kinematical ridges [21] were observed in *C* and $\tau _T$. Predictions in the kinematic vicinity of these exceptional points will require novel resummation approaches to overcome the associated instabilities of the fixed-order predictions. Moreover, at low values of the event shape variables, we observed the onset of large logarithmic corrections at each order in perturbation theory. These were particularly pronounced in $B_\gamma$. Resummation of these corrections is currently understood to next-to-leading logarithmic accuracy [22]. Aiming for a matching between fixed order and resummation in a form where the fixed-order expansion of the resummation formula reproduces all logarithmically enhanced terms up to NNLO (as was done for $e^+e^-$ event shapes [10, 12, 15]) will require two more logarithmic orders in the resummation.

To compare our parton-level predictions with hadron-level data, we used the dispersive model [28] to estimate the leading power correction effects from hadronization. The model is based on the study of two-point correlators which relate to the mean values of the event shape distributions. On the event shape distributions, additional assumptions must be made concerning the kinematical dependence of the power corrections. The power correction factors receive higher order contributions in the strong coupling constant, which we truncate to the same level as used in the fixed-order parton-level predictions.

Our resulting hadron-level predictions were compared to data from the H1 [23] and ZEUS [24] experiments. On the event shape distributions, we observe that inclusion of the NNLO corrections leads in general to an improved description of their kinematical shapes. Especially at medium and high $Q^2$, the NNLO corrections result in a substantial reduction of the scale uncertainties of the predictions, to the level of a few per cent. A similar reduction of scale uncertainty is also observed on the mean values. On these mean values, we observe a compensation between the positive NNLO corrections to the fixed-order parton-level predictions and the negative NNLO contributions to the power corrections, resulting in a relatively small net effect at NNLO. Our newly derived NNLO results yield predictions with scale uncertainties that are typically below the experimental errors of the available HERA data on event shape distributions. They motivate a full NNLO-based reanalysis of event shape distributions and mean values. This should be leading to an improved determination of $\alpha _s(M_Z)$ and $\alpha _0$, which was previously limited by the uncertainty on the NLO theory.

With high-resolution measurements of event shape distributions in deep inelastic scattering at a future electron-ion collider [52] or at the LHeC [53], our results will enable a broad spectrum of precision QCD studies, aiming for an improved understanding of its perturbative and non-perturbative aspects.

## Data Availability

This manuscript has associated data in a data repository. [Authors’ comment: The manuscript has associated data (annotated data tables for all predictions, with and without power corrections) which are stored as supplementary material by EPJC.]

## References

[1] Kluth S (2006). Rept. Prog. Phys..

[2] Newman P, Wing M (2014). Rev. Mod. Phys..

[3] Gehrmann-De Ridder A, Gehrmann T, Glover E W N, Heinrich G (2007). JHEP.

[4] Gehrmann-De Ridder A, Gehrmann T, Glover E W N, Heinrich G (2009). JHEP.

[5] A. Gehrmann-De Ridder, T. Gehrmann, E.W.N. Glover, G. Heinrich, Comput. Phys. Commun. **185**, 3331 (2014). arXiv:1402.4140

[6] Weinzierl S (2009). JHEP.

[7] Weinzierl S (2009). Phys. Rev. D.

[8] Del Duca V, Duhr C, Kardos A, Somogyi G, Szőr Z, Trócsányi Z, Tulipánt Z (2016). Phys. Rev. D.

[9] Gehrmann T, Glover EWN, Huss A, Niehues J, Zhang H (2017). Phys. Lett. B.

[10] Becher T, Schwartz MD (2008). JHEP.

[11] Chien Y-T, Schwartz MD (2010). JHEP.

[12] Abbate R, Fickinger M, Hoang AH, Mateu V, Stewart IW (2011). Phys. Rev. D.

[13] Monni PF, Gehrmann T, Luisoni G (2011). JHEP.

[14] Becher T, Bell G (2012). JHEP.

[15] Hoang AH, Kolodrubetz DW, Mateu V, Stewart IW (2015). Phys. Rev. D.

[16] S. Catani, M.H. Seymour, Nucl. Phys. **B485**, 291–419 (1997). arXiv:hep-ph/9605323. [Erratum: Nucl. Phys.B510,503(1998)]

[17] D. Graudenz, arXiv:hep-ph/9710244

[18] Nagy Z, Trocsanyi Z (2001). Phys. Rev. Lett..

[19] Antonelli V, Dasgupta M, Salam GP (2000). JHEP.

[20] Dasgupta M, Salam GP (2002). Eur. Phys. J. C.

[21] Dasgupta M, Salam GP (2002). JHEP.

[22] Dasgupta M, Salam GP (2004). J. Phys..

[23] H1 Collaboration, A. Aktas et al., Eur. Phys. J. **C46**, 343–356 (2006). arXiv:hep-ph/0512014

[24] ZEUS Collaboration, S. Chekanov et al., Nucl. Phys. **B767**, 1–28 (2007). arXiv:hep-ph/0604032

[25] T. Gehrmann et al., PoS RADCOR2017, 074 (2018). arXiv:1801.06415

[26] Currie J, Gehrmann T, Niehues J (2016). Phys. Rev. Lett..

[27] Currie J, Gehrmann T, Huss A, Niehues J (2017). JHEP.

[28] Dokshitzer YL, Marchesini G, Webber BR (1996). Nucl. Phys. B.

[29] Dasgupta M, Webber BR (1998). Eur. Phys. J. C.

[30] Dasgupta M, Webber BR (1998). JHEP.

[31] Hagiwara K, Zeppenfeld D (1989). Nucl. Phys. B.

[32] Berends FA, Giele WT, Kuijf H (1989). Nucl. Phys. B.

[33] Falck NK, Graudenz D, Kramer G (1989). Nucl. Phys. B.

[34] Glover EWN, Miller DJ (1997). Phys. Lett. B.

[35] Bern Z, Dixon LJ, Kosower DA, Weinzierl S (1997). Nucl. Phys. B.

[36] Campbell JM, Glover EWN, Miller DJ (1997). Phys. Lett. B.

[37] Bern Z, Dixon LJ, Kosower DA (1998). Nucl. Phys. B.

[38] Garland LW, Gehrmann T, Glover EWN, Koukoutsakis A, Remiddi E (2002). Nucl. Phys. B.

[39] Garland LW, Gehrmann T, Glover EWN, Koukoutsakis A, Remiddi E (2002). Nucl. Phys. B.

[40] Gehrmann T, Remiddi E (2002). Nucl. Phys. B.

[41] Gehrmann T, Glover EWN (2009). Phys. Lett. B.

[42] A. Gehrmann-De Ridder, T. Gehrmann, E.W.N. Glover, JHEP **09**, 056 (2005). arXiv:hep-ph/0505111

[43] Daleo A, Gehrmann T, Maitre D (2007). JHEP.

[44] Currie J, Glover EWN, Wells S (2013). JHEP.

[45] Currie J, Gehrmann T, Glover EWN, Huss A, Niehues J, Vogt A (2018). JHEP.

[46] H1 Collaboration, V. Andreev, et al., Eur. Phys. J. C **77**, 791 (2017). arXiv:1709.07251 10.1140/epjc/s10052-017-5314-7 PMC6956906 31997933

[47] D. Britzger et al., arXiv:1906.05303

[48] Britzger D, Currie J, Gehrmann T, Huss A, Niehues J, Žlebčík R (2018). Eur. Phys. J. C.

[49] Niehues J, Walker DM (2019). Phys. Lett. B.

[50] Gehrmann T, Huss A, Niehues J, Vogt A, Walker DM (2019). Phys. Lett. B.

[51] Catani S, Webber BR (1997). JHEP.

[52] Accardi A (2016). Eur. Phys. J. A.

[53] LHeC Study Group Collaboration, J.L. Abelleira Fernandez et al., J. Phys. **G39**, 075001 (2012). arXiv:1206.2913

[54] Catani S, Webber BR, Marchesini G (1991). Nucl. Phys. B.

[55] Davison RA, Webber BR (2009). Eur. Phys. J. C.

[56] Gehrmann T, Jaquier M, Luisoni G (2010). Eur. Phys. J. C.

[57] Dokshitzer YL, Lucenti A, Marchesini G, Salam GP (1998). JHEP.

[58] H1 Collaboration, C. Adloff et al., Eur. Phys. J. **C14**, 255–269 (2000). arXiv:hep-ex/9912052. [Erratum: Eur. Phys. J.C18,417(2000)]

[59] Moch S, Vermaseren JAM, Vogt A (2004). Nucl. Phys. B.

[60] Y.L. Dokshitzer, G. Marchesini, G.P. Salam, Eur. Phys. J. direct **1**, 3 (1999). arXiv:hep-ph/9812487

[61] H1 Collaboration, V. Andreev et al., Eur. Phys. J. **C77**, 215 (2017). arXiv:1611.03421

